# Editorial: Hormonal imbalance-associated oxidative stress and protective benefits of nutritional antioxidants

**DOI:** 10.3389/fendo.2024.1368580

**Published:** 2024-02-09

**Authors:** Dipak Kumar Sahoo, Luna Samanta, Kavindra Kumar Kesari, Sutapa Mukherjee

**Affiliations:** ^1^ Department of Veterinary Clinical Sciences, College of Veterinary Medicine, Iowa State University, Ames, IA, United States; ^2^ Department of Zoology, Ravenshaw University, Cuttack, Odisha, India; ^3^ Research and Development Cell, Lovely Professional University, Phagwara, Punjab, India; ^4^ Department of Applied Physics, Aalto University, Espoo, Finland; ^5^ Department of Zoology, Visva-Bharati University, Bolpur, West Bengal, India

**Keywords:** endocrine disorders, oxidative stress, redox imbalance/homeostasis, hormone receptors, mitochondrial dysfunction, antioxidants, ROS

The intricate interplay exists among the endocrine system, redox equilibrium, and oxidative stress (OS) in various vital biological processes encompassing fertilization, embryonic development, somatic growth, aging, and pathophysiological conditions (**Figure 1**). Understanding the relationship between hormonal conditions, redox state, and OS in living systems is an intricate challenge (1, 2). Reaching a unified conclusion becomes more challenging when certain hormones display oxidant capabilities while others possess antioxidant characteristics. In comparison to the antioxidative properties exhibited by hormones such as melatonin, estrogen, progesterone, and insulin, hormones such as thyroid hormone, catecholamines, and corticosteroids have the ability to augment the production of free radicals and OS as a consequence of disequilibrium in redox homeostasis (1, 2). Aerobic cells possess a proficient antioxidant defense mechanism that serves to counteract the deleterious impact of reactive oxygen species (ROS) through the maintenance of redox homeostasis (3). The elucidation of hormonal fluctuations and the examination of the mechanisms underlying the effects of antioxidants hold promise for the advancement of novel therapeutic approaches targeting disorders associated with hormonal dysregulation (**Figure 1**).

**Figure 1:**
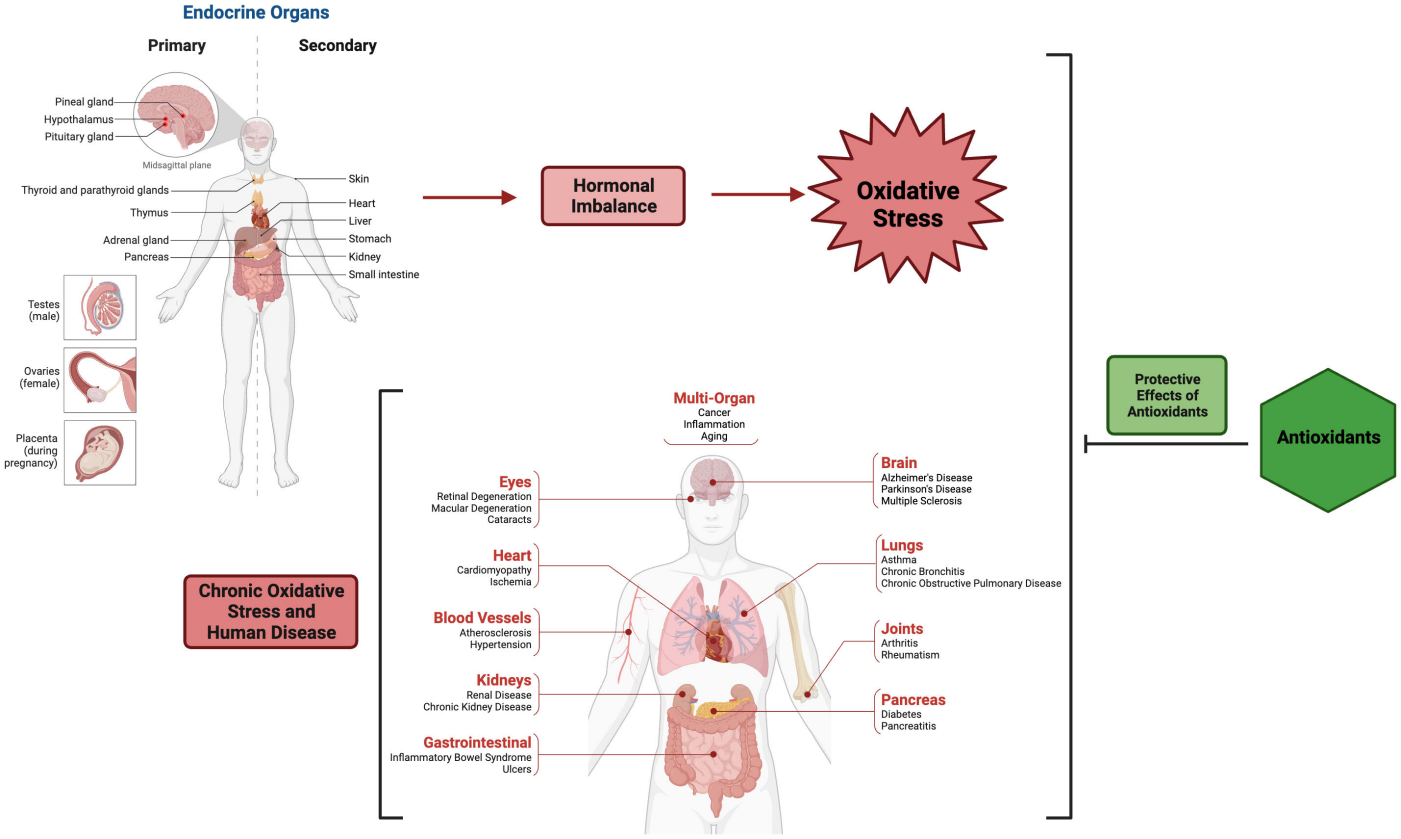
Role of antioxidants in hormonal imbalance-associated oxidative stress and various pathological conditions. The figure was generated using BioRender (www.biorender.com; accessed on 10^th^ Jan 2024).

Various plant-based compounds, such as alkaloids, flavonoids, phenolic acid, and coumarin, have been shown to possess anti-diabetic and antioxidative effects (**Figure 1**) (4, 5). The review article by Chhabria et al. explores the potential of plant-based Dipeptidyl peptidase (DPP)-IV inhibitors in combating OS in diabetes-related pathological conditions and also delves into the study of the creation of polyherbal formulations and nanophytomedicines to regulate incretin activity. The review by Nelson et al. presents multiple preclinical and clinical studies demonstrating the strong anti-colon cancer effects of alkaloids derived from medicinal plants and phytocompounds. These alkaloids have shown minimal toxicity towards normal cells. Additionally, the studies indicate that various alkaloids can induce apoptosis in colon cancer cells by targeting different cellular components, such as hormones and growth factors, which are involved in metastasis, angiogenesis, proliferation, and invasion. This review offers a detailed account of each alkaloid that has undergone clinical trials, either alone or in combination with other drugs, and also discusses different classes of phytochemicals that can induce cell death in various types of cancers, including colon cancer. Sea buckthorn and its bioactive ingredients show promise in addressing gynecological issues like uterine inflammation and endometriosis and alleviating symptoms of vulvovaginal atrophy in postmenopausal women (Mihal et al.). The polyphenolic flavonoids found in sea buckthorn have various health benefits and exhibit antioxidant, anti-inflammatory, and anti-cancer properties. They also play a role in promoting healthy ovarian cell proliferation, regulating cell death, and hormone release. Additionally, sea buckthorn may help reduce the risk of ovarian cancer by promoting apoptosis and regulating estrogen release.

The study by Kolesarova et al. explores the promising potential of pomegranate peel extract (PPE) in the prevention and/or therapy of ovarian cancer. A brief treatment with PPE suppressed human adenocarcinoma cell line metabolic activity and elevated the expression of cyclin-dependent kinase 1. Also, the administration of PPE resulted in a reduction in the secretion of growth factors, specifically TGF-β2 and EGF. Additionally, the expression of their respective receptors, TGFBR2 and EGFR, was also diminished. The research review by Kohut et al. delves into the effects of grapeseed extract and grape polyphenols on female reproductive processes. Research has shown that grape extract and its polyphenols, like resveratrol, proanthocyanidin B2, and delphinidin, have the potential to impact female reproductive health. These compounds can regulate various signaling pathways involved in reproductive hormones, steroid hormone receptors, oxidative stress, inflammation, apoptosis, and cell growth. The significance of these compounds in the treatment of ovarian cancer, ovarian ischemia, polycystic ovary syndrome, age-related reproductive insufficiency, or menopausal syndrome has been suggested. Grapeseed extracts and/or proanthocyanidin B2 and delphinidin may have an impact on developmental capacity, ovarian steroidogenesis, and oocyte maturation, although these effects may occur at varying regulatory levels.

Utilizing the potency of dietary antioxidants has promise in mitigating OS resulting from adrenal hormone imbalance (Patani et al.). This review explores the benefits of different nutritional antioxidants, including selenium, zinc, polyphenols, coenzyme Q10, vitamin C, vitamin E, carotenoids, and probiotics, in reducing the negative impacts of OS caused by abnormalities in adrenal hormone levels. Various studies have reported the importance of nutritional antioxidants in preserving a healthy balance of redox homeostasis in various thyroid pathologies. Several research studies have indicated a favorable correlation between beta-carotene levels and thyroid function. However, other investigations have not observed a notable impact. The review article by Far et al. explores the interactions between beta-carotene/retinol and thyroid hormones, as well as the results of clinical trials investigating the relationship between beta-carotene intake and thyroid hormone levels. The review article by Macvanin et al. offers insights into the role of nutritional antioxidants in maintaining a healthy balance of redox homeostasis in different thyroid pathologies. These pathologies include the development of diseases like goiter, thyroid cancer, or thyroiditis. New findings regarding the link between the thyroid gland and gut microbiome were also explored, and the impact of probiotics with antioxidant properties on thyroid diseases was also assessed.

Natural mating can trigger alterations in the activity of female genes that control antioxidant enzymes crucial for the survival of sperm during their transport, primarily influenced by estrogen present in the bloodstream and semen. The study conducted by Álvarez-Rodríguez et al. reveals interesting findings regarding changes in the reproductive tract following mating, including a decrease in the expression of estrogen and progesterone receptors, as well as an increase in superoxide dismutase 1, glutaredoxin 3, and peroxiredoxin 1 and 3 that may play a role in preventing OS in the region near the sperm reservoir at the utero-tubal junction. However, Multiple studies highlight the significance of hormones, ROS generation, and their impact on male fertility. A systematic review and meta-analysis study conducted by Ebrahimi et al. analyzed 20 toxic materials and found that melatonin therapy improved reproductive hormonal panel, testicular histopathological characteristics, and tissue markers of oxidative stress. Research suggests that melatonin has antioxidant properties and could potentially safeguard testicular tissue against the harmful effects of toxic substances. Melatonin therapy also had positive effects on testicular health in male rodents with various types of testicular injuries (Ebrahimi et al.). Another systematic review and meta-analysis found evidence supporting the protective effects of melatonin against anti-cancer stressors in rodent testicular tissue. The meta-analysis revealed significant improvements in various outcomes with melatonin therapy, including enhancements in sperm quantity and quality, as well as improvements in the serum levels of reproductive hormones (testosterone and Follicle-Stimulating Hormone). Additionally, melatonin therapy decreases tissue markers of oxidative stress, such as testicular tissue malondialdehyde and caspase-3 activity. At the same time, it increases glutathione and total antioxidant capacity along with superoxide dismutase, catalase, and glutathione peroxidase activities (Ebrahimi et al. **)**.

Henceforth, antioxidant supplements (5, 6) have emerged as a subject of discourse in contemporary times, particularly in light of their purported efficacy in addressing various pathological conditions, including endocrine disorders, and serving as adjunctive modalities to enhance conventional therapeutic interventions.

## Author contributions

DS: Conceptualization, Methodology, Project administration, Resources, Supervision, Writing – original draft, Writing – review & editing. LS: Conceptualization, Writing – review & editing. KK: Writing – review & editing. SM: Writing – review & editing.
